# Effect of university students’ sedentary behavior on stress, anxiety, and depression

**DOI:** 10.1111/ppc.12296

**Published:** 2018-05-24

**Authors:** Eunmi Lee, Yujeong Kim

**Affiliations:** ^1^ Department of Nursing Hoseo University Asan‐si Korea

**Keywords:** anxiety, depression, sedentary behavior, stress

## Abstract

**Purpose:**

We identified the effect of sedentary behavior on stress, anxiety, and depression among Korean university students.

**Design and Methods:**

Data were collected from 244 students using self‐reported sitting time, the Perceived Stress Scale, the Beck Anxiety Inventory, and the Center for Epidemiological Studies‐Depression Scale.

**Findings:**

Mean sitting time was 7.96 h per day. As sitting hours increased, university students’ stress, anxiety, and depression significantly increased despite controlling for sex, economic level, body mass index, underlying disease, and health self‐management.

**Practical Implications:**

Intervention programs that reduce sedentary behavior and improve physical activity and mental health for university students are necessary.

## 1 INTRODUCTION

### 1.1 Effect of university students’ sedentary behavior on stress, anxiety, and depression

Various changes occur during university as one transitions from the intense competition of entrance exams to increased opportunities for drinking, smoking, and job search stress. In the context of the life cycle, this is a period when one's lifestyle is relatively flexible compared to that of adulthood, and it is likely to undergo corrective change.^1^ Therefore, university is an important stage for developing healthy habits. However, physical activity is lacking for many Korean university students, which does not increase after high school and is half the level of their American counterparts.^2^ University students who are not majoring in physical education rarely engage in physical activity in their subject area.^3^ Moreover, with the growing use of smartphones, playing computer games, and watching TV, the time spent sitting down is steadily increasing compared to the time spent engaging in physical activities.^3^

Convenience has increased due to technological developments; however, reduced physical activity comes with a higher morbidity of various diseases. According to the World Health Organization,^4^ more than 60% of the world's population does not even engage in moderate physical activity (i.e., 30 min per day). The need for physical activity and the effect of physical activity on physical and mental health have long been confirmed; however, the recent increase in sedentary behaviors due to jobs, computer games, smartphones, and television warrants attention from a health management perspective.^5^

Sedentary behaviors refer to those involving decreased energy expenditures during waking hours and includes both sitting and lying down.^6^ Only a limited number of studies have measured university students’ sitting time, with no such studies available in Korea. Specifically, American university students spend 4.2 h per day sitting down on average.^7^ In addition, most studies have focused on adults; in a study that monitored the sitting time of adults in 20 countries, the daily average was 5.8 h,^8^ and adults in the United States,^9^ Canada,^10^ and the United Kingdom^11^ spent 9–11 h per day sitting down, comprising 55–70% of their waking hours.

Maintaining a lifestyle that revolves around sitting down can lead to health risks in adulthood. Recent studies show that individuals with increased sedentary behavior have higher risks of obesity, type 2 diabetes, bone density loss, cardiovascular disorders, and endometrial cancer compared to those who sit less.^5^, ^12^, ^13^, ^14^

Although several studies have addressed sedentary behavior, physical activity, and physical health problems, few studies have examined sedentary behavior and mental health.^4^, ^5^ Among such studies, a study from Australia found that sedentary behavior was not correlated with mental health‐related quality of life.^15^ Another Australian study reported significantly higher psychological distress among office workers who spent more than 6 h sitting down for work compared to those who did not.^16^ Moreover, in a study conducted with university students in Spain, students who spent more than 42 h per week sitting down had a 31% higher risk of mental disorders compared to students who did not.^17^

Importantly, suicide mortality in South Korea ranks first among the Organization for Economic Cooperation and Development (OECD) countries since 2003 (28.7 per 100,000 people).^18^ Among deceased individuals aged 20–29 years, 45.5% are lost due to suicide.^19^ Moreover, university students’ stress, anxiety, and depression were found to have a direct impact on their suicide rate.^20^

It is important to maintain and habituate a healthy lifestyle during university and to continue such healthy habits in adulthood. Therefore, it is necessary to determine how long university students’ sedentary behavior lasts and how it affects their mental health. Consequently, we examined the effect of university students’ sedentary behavior on their stress, anxiety, and depression per their demographic characteristics.

## 2 METHODS

### 2.1 Sampling and data collection

Participants comprised students from two universities in South Korea. Data were collected from April 3 to 14, 2017. The researcher explained the study's objectives and questionnaire items to students enrolled in chapel class; then, the questionnaires were submitted in a collection box. Chapel classes were chosen because they comprised all liberal arts classes, regardless of participant major. The survey took approximately 10–15 min to complete. Questionnaires were distributed to 300 participants and 282 were collected; however, 244 were used for final analysis after excluding 38 that were incomplete.

### 2.2 Instruments

#### 2.2.1 Sedentary behavior

Sedentary behavior refers to activities that do not increase energy expenditure substantially above the resting level (1.0∼1.5 METs) and includes activities such as sleeping, sitting, lying down, watching TV, and other forms of screen‐based entertainment.^12^ In this study, we measured sitting time per university students’ life characteristics. Daily average sedentary behavior was investigated for weekdays and weekends separately. Times for each were calculated as weighted averages of 5/7 for weekdays and 2/7 for weekends.

#### 2.2.2 Stress

The Korean version of the Perceived Stress Scale (PSS), which was developed by Cohen et al.^21^ was employed in this study. Park and Seo^20^ verified its validity and reliability with Korean university students. The PSS is a self‐report scale that was categorized into two factors: negative perception and positive perception. Negative perception is perceived to be beyond one's control. Positive perception is the measurement of how well one predicts, controls, and copes with everyday events. The PSS measured 10 items across these two categories (5 items in each category) with a 5‐point Likert scale. Higher scores indicate a higher level of perceived stress. The PSS is popular, easy to understand, and quick to complete. Cronbach's α in Park and Seo^20^ was 0.82; Cronbach's α in this study was 0.85.

#### 2.2.3 Anxiety

Anxiety was measured with the Beck Anxiety Inventory (BAI), a self‐report scale that was developed by Beck et al.^22^ and standardized into Korean by Kwon.^23^ The BAI is one of the most used and easy to understand. The questionnaire consists of 21 items including cognitive, emotional, and physical dimensions of anxiety. Each item was measured via a 4‐point Likert scale that ranged from “*strongly disagree* (0)” to “*strongly agree* (3).” The total score ranged 0–63 points. The Cronbach's α in this study was 0.93.

#### 2.2.4 Depression

Depression was measured using the Center for Epidemiological Studies‐Depression Scale (CES‐D). The CES‐D is a self‐report scale that was developed by Radloff^24^ to study depression dynamics in a group of normal people. It is a 20‐item measure that asks participants to rate how often over the past week they experienced symptoms associated with depression such as restless sleep, poor appetite, and feeling lonely. Symptoms were measured using a 4‐point Likert scale (0–3 points). Scores range from 0 to 60, with high scores indicating greater depressive symptoms. This study used a Korean version of the scale adapted by Chon and Rhee,^25^ which was measured with a 5‐point Likert scale, with higher scores indicating higher levels of depression. The CES‐D is widely used, easy to understand, and quick to complete. The Cronbach's α for this study was 0.93.

### 2.3 Ethical considerations

The study was approved by the university's Institutional Review Board (no. 1044396‐201703‐HR‐051‐01). Ethical issues including plagiarism, informed consent, misconduct, data fabrication and/or falsification, double publication and/or submission, and redundancy were monitored by the author.

### 2.4 Data analysis

Collected data were analyzed using IBM SPSS 20.0 statistical software. A frequency analysis was conducted for participants’ demographic characteristics. To confirm the applicability of the control variable, an independent samples *t*‐test and a one‐way analysis of variance (ANOVA) were conducted when comparing differences in stress, anxiety, and depression per demographic characteristics. A simple logistic regression analysis was conducted to examine the effect of sedentary behavior on stress, anxiety, and depression.

## 3 RESULTS

### 3.1 Levels of sedentary behavior, stress, anxiety, and depression

Levels of sedentary behavior, stress, anxiety, and depression are shown in Table 1.

**Table 1:** Descriptive statistics by sedentary behavior, stress, anxiety, and depression

Variable	Mean	*SD*	Min	Median	Max
Sedentary behavior (hour)	7.96	3.35	0.49	7.57	18.00
Sedentary behavior during the weekdays	8.40	3.60	0.67	8.00	18.00
Sedentary behavior during the weekends	6.80	3.95	0.05	6.00	18.00
Stress	2.91	0.64	1.10	2.87	4.90
Anxiety	0.58	0.51	0.00	0.55	2.76
Depression	2.58	0.73	1.00	2.49	4.60

*Note*. Max, maximum; Min, minimum; *SD*, standard deviation.

### 3.2 Differences in stress, anxiety, and depression per demographic characteristics

Differences in stress levels according to demographic characteristics were examined to study the relationship between control variables and dependent variables (Table 2). Results showed statistically significant differences for sex, underlying disease (physical diseases such as atopic dermatitis, rhinitis, and asthma), and self‐management for health. For sex, women were more stressed than men were. Those with an underlying disease were more stressed than those without. Self‐management for health was shown to lower stress. An examination of the differences in anxiety according to demographic characteristics revealed significant differences for sex and self‐management for health. Women had higher anxiety than men did. Self‐management for health was shown to lower anxiety. Examining the differences in depression according to demographic characteristics revealed significant differences for sex, body mass index (BMI), underlying disease, and self‐management for health. Women displayed more depression than men did. Obese participants showed higher depression than the others did. Participants with an underlying disease were more depressed than those who were not. Self‐management for health was found to lower depression (Table 2).

**Table 2:** Stress, anxiety, and depression per participants’ demographic characteristics (*N* = 244)

			Stress (1–5)		Anxiety (0–3)		Depression (1–5)	
Variable	Classification	*n* (%) or Mean ± *SD*	Mean ± *SD*	*t*, *F* (*p*)	Mean ± *SD*	*t*, *F* (*p*)	Mean ± *SD*	*t*, *F* (*p*)
Sex	Male	49 (20.1)	2.68 ± 0.62	–2.857	0.40 ± 0.34	–3.824	2.30 ± 0.63	–3.172
	Female	195 (79.9)	2.97 ± 0.64	(0.005)	0.63 ± 0.54	(<0.001)	2.66 ± 0.74	(0.002)
Living with parents	Yes	73 (29.9)	2.89 ± 0.69	–0.395	0.56 ± 0.55	–0.501	2.59 ± 0.78	0.106
	No	171 (70.1)	2.92 ± 0.62	(0.693)	0.59 ± 0.50	(0.617)	2.58 ± 0.71	(0.916)
BMI (kg/m^2^)	Underweight (<18.5)^a^	42 (16.7)	3.07 ± 0.63		0.66 ± 0.56		2.73 ± 0.64	
	Normal (≤18.5–22.9)^b^	154 (65.1)	2.84 ± 0.63	1.790	0.54 ± 0.51	2.047	2.48 ± 0.76	2.801
	Overweight (≤23–24.9)^c^	27 (9.3)	3.09 ± 0.74	(0.150)	0.76 ± 0.58	(0.108)	2.90 ± 0.71	(0.041)
	Obesity (≥25.0)^d^	21 (8.8)	2.82 ± 0.85		0.41 ± 0.36		2.44 ± 0.74	b,d < c
Underlying disease	No	198 (80.0)	2.87 ± 0.64	–1.963	0.55 ± 0.49	–1.807	2.53 ± 0.73	–2.128
	Yes	46 (20.0)	3.08 ± 0.66	(0.051)	0.71 ± 0.63	(0.720)	2.79 ± 0.71	(0.034)
Perceived economic status	Very high	6 (2.5)	2.60 ± 0.62		0.61 ± 0.67		2.43 ± 0.82	
	High	33 (13.6)	2.77 ± 0.62		0.63 ± 0.69		2.41 ± 0.76	
	Moderate	150 (62.0)	2.93 ± 0.66	–1.854	0.56 ± 0.48	–0.364	2.61 ± 0.75	–1.052
	Low	49 (20.2)	3.03 ± 0.62	(0.065)	0.59 ± 0.48	(0.716)	2.66 ± 0.66	(0.294)
	Very low	4 (1.7)	2.73 ± 0.48		1.01 ± 0.48		2.30 ± 0.65	
Self‐management for health	Not at all^a^	39 (16.0)	3.24 ± 0.62		0.85 ± 0.57		2.95 ± 0.74	
	Little^b^	123 (50.4)	2.96 ± 0.59	–4.046	0.58 ± 0.46	–3.316	2.70 ± 0.65	–5.344
	A little^c^	73 (29.9)	2.64 ± 0.63	(<0.001)	0.44 ± 0.49	(0.001)	2.23 ± 0.72	(<0.001)
	Very much^d^	9 (93.7)	3.15 ± 0.87	c < a,d	0.67 ± 0.78	c < a	2.42 ± 0.66	c < a

*Note*. BMI, body mass index; *SD*, standard deviation.

### 3.3 Effect of sedentary behavior on stress, anxiety, and depression

A simple logistic regression model was conducted to examine the effect of sedentary behavior on stress, anxiety, and depression (Table 3). Model 1 was a simple model using sedentary behavior as the independent variable and stress, anxiety, and depression as dependent variables. Model 2 used sex, perceived economic status, BMI, underlying disease, and self‐management for health as control variables. Sedentary behavior during weekdays and weekends were found to have a significant effect on stress, anxiety, and depression in all models.

**Table 3:** Effect of sedentary behavior on stress, anxiety, and depression

	Stress				Anxiety				Depression			
	Model 1^a^		Model 2^b^		Model 1^a^		Model 2^b^		Model 1^a^		Model 2^b^	
Variable	Slope	t (*p*)	Slope	t (*p*)	Slope	t (*p*)	Slope	t (*p*)	Slope	t (*p*)	Slope	t (*p*)
Sedentary behavior (hour)	0.030	2.305 (0.022)	0.032	2.270 (0.024)	0.025	2.351 (0.020)	0.028	2.414 (0.017)	0.041	2.818 (0.005)	0.046	2.981 (0.003)
Sedentary behavior during the weekdays	0.024	2.036 (0.043)	0.028	2.135 (0.034)	0.022	2.240 (0.026)	0.026	2.434 (0.016)	0.030	2.223 (0.027)	0.037	2.579 (0.011)
Sedentary behavior during the weekends	0.025	2.267 (0.024)	0.023	1.837 (0.068)	0.019	2.125 (0.035)	0.018	1.780 (0.077)	0.042	3.419 (0.001)	0.039	2.912 (0.004)

*Note*. ^a^Simple regression analysis; ^b^Control variable: sex, perceived economic status, body mass index, underlying disease, and self‐management for health.

## 4 DISCUSSION AND CONCLUSION

This study was conducted to determine the degree and relationship between sedentary behavior, stress, anxiety, and depression in Korean university students. First, Korean university students in this study spent nearly double the time sitting than U.S. students in a past study did,^7^ and more than the daily average (5.8 h) reported among adults in 20 countries.^8^

A clinical and public health guideline for sedentary behavior is not yet established; however, some studies show the negative effects of long sitting hours, such as a 18–45% mortality rate, compared to those with shorter sitting times.^26^ Problematically, Korean university students experience a reduction in physical activity in adolescence compared to adolescents in different countries due to factors such as entrance exam preparation, and the prior level of physical activity is not recovered when they reach university.^27^ Furthermore, many Korean university students face problems such as obesity, anemia, and gastrointestinal diseases, which require active health management including physical activity.^28^

Second, compared to other studies using the same tool, the stress level of Korean university students was higher than the levels among American university students (1.75–1.97)^29^, ^30^ and Mexican university students (1.52).^31^ Anxiety levels were also higher than those found among Spanish university students, which were measured in another study using the same tools.^32^ In addition, depression levels were higher than those found among university students from China and Hong Kong.^33^ Stress, anxiety, and depression are major mental health problems experienced not only by Korean students but also by university students around the world.^34^ These inhibit students’ academic performance, social relationships, and adjustment to university life. In addition, the added pressure from university life has a latent impact on their employment.^35^ Moreover, university students’ stress, anxiety, and depression were also found to have a direct impact on their suicide rates.^37^, ^38^ However, even university students around the world have difficulties asking for help from the people around them or seeking specialists’ advices regarding mental health problems.^38^ Therefore, universities and families need to pay special attention to the mental health of students, and local communities and on‐campus counsellors should promptly and actively intervene to ensure that vulnerable students can effectively overcome and cope with stress and emotional crises.

Third, this study showed that increased sedentary behavior elevated stress, anxiety, and depression despite controlling for sex, perceived economic status, BMI, disease, and self‐management for health. Research on the correlation between university students’ sedentary behavior and mental health is limited; however, a 6‐year cohort study on university graduates in Spain revealed that participants who were seated for over 42 h per week were 1.3 times more likely to experience mental disorders such as stress, anxiety, and depression compared to participants who sat 10.5 h per week.^17^ On the other hand, one study showed that while sedentary behavior was significantly related with anxiety and depression, no significant correlation was found with stress.^39^ In addition, another study reported no correlation between sedentary behavior and mental health,^15^ contradicting the results of this study. Additionally, participants with more than 6 h of seated working were found to have a significantly higher level of psychological distress compared to those who had less.^40^ Another study found that stress, anxiety, and depression were significantly higher in participants who spent their leisure time sitting down.^15^ Therefore, additional research needs to differentiate sedentary behavior into working sitting and nonworking sitting (e.g., leisure sitting, computer sitting, smartphone sitting, TV sitting, transporting sitting, etc.). Furthermore, researchers should examine the correlation between sedentary behavior and mental health in longitudinal studies. Lastly, while studies are promoting art‐based, psychoeducational, cognitive, behavioral, and mindfulness‐based techniques as intervention methods to reduce university students’ stress and anxiety,^16^ consideration for intervention methods that promote physical activity is also suggested.

### 4.1 Recommendations for future research

Based on this study, future research should address the following: first, a varied sampling range across different regions should be employed; second, the effects related to type or purpose of sedentary behavior on mental health should be identified and the type of sedentary behavior could be classified into working sitting and nonworking sitting (e.g., leisure sitting, computer sitting, smartphone sitting, TV sitting, transporting sitting, etc.); third, the effect of personality and coping skills on sedentary behavior should be examined; and, lastly, since this study was cross‐sectional, causality and direction of relationships could not be determined. Therefore, prospective cohort studies that address the effects of physical and mental health, workplace adaptability, and job satisfaction are also recommended.

### 4.2 Implications for nursing practice

Stress, anxiety, and depression are serious mental health problems among college student groups. The sedentary behavior of college students was found to be significantly related to their psychological problems in the present study. However, until now, research has rarely been conducted to identify the mechanisms or mediating variables that explain the relationship between sedentary behavior and stress, anxiety, and depression. Based on the social withdrawal hypothesis, some studies have explained that the more people watch TV or use computers or the Internet, the more their sedentary behavior increases, resulting in decreased social interactions that result in turn in an increased risk of mental health problems.^41^ Some other studies have speculated that replacing physical activities that promote mental health with sedentary behavior could increase the possibility of causing psychological problems.^42^, ^43^ Accordingly, researchers and psychiatric nurses need to investigate further the mechanism between sedentary behavior and stress, anxiety, and depression in research and clinical fields. The findings of such studies can present useful information for policymakers and healthcare professionals including psychiatric nurses to design effective mental health improvement programs.

Universities are key settings for both education and health; therefore, they must create an environment where students can exhibit a healthy lifestyle. South Korean college students are, however, in a situation in which they are bound to engage in life patterns that involve much sedentary behavior due to competitive grade‐getting, unemployment problems, and lack of leisure. Therefore, college mental health services should educate them about the signs and symptoms of mental problems and effective coping methods and operate campus intervention programs that reduce sedentary behavior, increase physical activity, and enhance students’ mental health. Students with severe levels of stress, anxiety, and depression should especially receive counseling for their psychological problems and have easy access to mental health clinics through in‐school counseling centers.

Psychiatric nurses can help students express their psychological difficulties and help them change their behaviors and lifestyles. In addition, the present study can help psychiatric nurses present evidence in developing psychotherapeutic intervention models and practical interventions.

Preventive strategies include implementing technological devices, more breaks in class and while studying, using sit‐stand desks, and increasing campus walkability. Developing these interventions could promote a deeper understanding of psychosocial barriers and motivations behind why college students engage in prolonged sedentary behaviors. Additional longitudinal research related to the effects of these intervention programs should be implemented.

## CONFLICT OF INTEREST STATEMENT

The authors report no actual or potential conflicts of interest.
